# Progress in Ophthalmology

**Published:** 1904-01-02

**Authors:** 


### Progress in Ophthalmology.

**Operations for Conical Cornea.**[1]—Like the numerous operations that have been devised for entropion the number of operations tried for the improvement of conical cornea is legion, and this number may in a way be taken as a gauge of their success. Mr. Stanford Morton, however, speaks very highly of his method to which, he says, he was led by reading the successes of Higgins with a similar method. The earlier cauterisation without and with perforation of the cornea showed great success immediately afterwards, and during the time the eyes were bandaged and kept under eserine, but sooner or later the faulty vision returned, with, in addition, a central corneal opacity to make it worse. The operation Morton favours may be described in his own way thus: "The apex of the cone is transfixed by a long, narrow, and stiff Graefe knife whose edge is turned forwards and a little to the right. The knife is pushed steadily onwards until it cuts its way out through the cornea to the right of the apex of the cone, thus forming one edge to the elliptical piece to be excised. The flap thus formed is held up by its free edge with fine forceps, the knife being then passed underneath the points of these forceps, cuts out on their left side through the corneal tissue, and so completes the excision of an elliptical piece, about 2 m.m. by 1 m.m., containing the apex of the cone." Excision of the apex may be performed either in the vertical or horizontal meridian, or in the meridian of least curvature. There is not, perhaps, very much choice between the vertical and horizontal incisions, though, possibly, with the former, the wound is more under cover of the lids, and, therefore, less disturbed than in a horizontal incision which might be just in the line of the palpebral aperture. From a perusal of his notes, he says, it will be seen that operations for conical cornea are not devoid of risks, and should be undertaken only when other means fail to give the desired vision. The complications which occurred were: Anterior synechiæ, iritis, and posterior synechia, increased tension, and hypopyon. The history of subsequent vision is very good, two of the 13 cases having 6 6 and three 6·9. Two of the 6·9 eyes were under observation for 20 years. This does away with any doubts as to the faulty sight recurring.

**Some Forms of Ophthalmia.**—Treacher Collins,[2] in a lecture on this subject, says in the treatment of gonorrhœal conjunctivitis in the adult. Cases we have to deal with from the very early stages will occasionally, in spite of all our efforts, get ulceration of the cornea, which may go on to perforation and destruction of the eye. Prevention of such an affection is therefore of the greatest importance. He comments on the necessity of warning the patients and their friends of the danger of getting any of the discharge into the eye. "In the early stages of gonorrhœal ophthalmia, when there is considerable swelling of the lids and conjunctiva without any discharge, strong caustic application should not be employed. At this stage it is best to keep ice pads constantly applied, by which means the amount of swelling will often be considerably reduced." So far so good. This is the orthodox treatment, and is no doubt the result of observation by generations of ophthalmic surgeons; but if the danger of sloughing cornea be the bugbear of the disease, why put ice on it to make its destruction more certain? Why not hot fomentations? Surely a cornea is more likely to slough when it is kept as near freezing-point as possible than if it were kept at a more genial temperature; this has always been an anomaly to our mind, and in our treatment we have always employed and taught the advantage of heat over cold in this distressing malady and we are pleased to think without any untoward results.

[1] Brit. Med. Jour., Sept. 26, 1903. [2] Clin Jour., July 29, 1903.

*(To be continued.)*

